# Impact of Adjuvant Therapy on Survival in Surgically Resected Limited-Stage Small Cell Lung Cancer

**DOI:** 10.3389/fonc.2021.704517

**Published:** 2021-09-23

**Authors:** Di Li, Chaoqiang Deng, Qiang Zheng, Fangqiu Fu, Shengping Wang, Yuan Li, Haiquan Chen, Yang Zhang

**Affiliations:** ^1^ Department of Thoracic Surgery and State Key Laboratory of Genetic Engineering, Fudan University Shanghai Cancer Center, Shanghai, China; ^2^ Institute of Thoracic Oncology, Fudan University, Shanghai, China; ^3^ Department of Oncology, Shanghai Medical College, Fudan University, Shanghai, China; ^4^ Department of Pathology, Fudan University Shanghai Cancer Center, Shanghai, China; ^5^ Department of Radiology, Fudan University Shanghai Cancer Center, Shanghai, China

**Keywords:** small cell lung cancer (SCLC), adjuvant chemotherapy (ACT), adjuvant chemoradiotherapy (ACRT), overall survival (OS), prognosis

## Abstract

**Background:**

Data on efficacy of adjuvant therapy for surgically resected small cell lung cancer are scant. This study was determined to reveal the survival benefits of different adjuvant treatment modalities for limited-stage small cell lung cancer patients following surgical resection.

**Methods:**

Data of patients with histologically confirmed small cell lung cancer after surgical resection were collected from November 2006 to June 2019. Survival analyses were calculated by Kaplan–Meier method, with log-rank test to evaluate statistical significance. Prognostic factors were identified by multivariate analysis using cox proportional hazards model. Further survival analysis and cox regression analysis stratified by clinicopathologic features were conducted to evaluate the survival benefits of different adjuvant treatment modalities.

**Results:**

In total, 153 out of 157 patients were analyzed. Multivariate analysis showed male sex, lymph node metastasis, residual tumor, VPI and non-adjuvant therapy were independently associated with poor prognosis. Subgroup analyses revealed both adjuvant chemotherapy and adjuvant chemoradiotherapy were significantly associated with superior survival for stage pT2-4 (HR=0.176, 95%CI:0.053-0.578, p=0.004; and HR=0.115, 95%CI:0.033-0.405, p=0.001) and pure SCLC patients (HR=0.182, 95%CI:0.067-0.494, p=0.001; and HR=0.181, 95%CI:0.071-0.465, p<0.001). For pN0 patients, adjuvant chemotherapy was associated with better survival (HR=0.219, 95%CI:0.054-0.891, p=0.034), while adjuvant chemoradiotherapy was associated with improved survival for pN+ patients (HR=0.324, 95%CI:0.138-0.760, p=0.010).

**Conclusions:**

For patients without pathologic lymph node metastasis, there is a survival benefit with adjuvant chemotherapy. However, for patients with pathologic lymph node metastasis, adjuvant chemoradiotherapy might achieve a significant survival benefit. Further prospective studies are needed to validate the results.

## Introduction

Lung cancer accounts for 12% of new cases of cancer worldwide and is the leading cause of cancer-related deaths (1). Small cell lung cancer (SCLC) is increasing by more than 180,000 cases per year, accounting for approximately 15% of all newly diagnosed cases of lung cancer worldwide (2–4). Although rare cases have been reported among non-smokers, almost all SCLC patients are current or former smokers (5, 6). In countries with high smoking rates, such as China, the incidence of SCLC is expected to remain elevated (3). SCLC progresses rapidly, and more than 70% of SCLC patients already have lymph nodes diseases or distant metastases at the time of diagnosis (7).

Platinum-based chemotherapy combined with radiotherapy remains the predominant treatment modality for SCLC patients (8–11). According to the National Comprehensive Cancer Network guidelines, surgical resection is recommended for early-stage SCLC patients (cT1-2N0M0) (9, 11). Nevertheless, due to the highly aggressive nature of SCLC, the number of operable SCLC patients is quite small. Consequently, data on efficacy of adjuvant therapy for resected SCLC are scarce. Previous studies have focused on the survival benefit of patients with SCLC after surgical resection, and the results have shown that patients with surgical resection have favorable survival (12–15). In recent years, there have been several studies concerning the benefits of specific adjuvant treatment modalities (16–22). However, few studies have comprehensively analyzed the benefits of different adjuvant treatment modalities for patients with surgically resected SCLC.

In this study, therefore, we analyzed the survival of SCLC patients with surgical resection and investigated the prognostic factors of these patients. Furthermore, we evaluated the impact of adjuvant chemotherapy and adjuvant chemoradiotherapy on the overall survival stratified by clinicopathologic features.

## Patients and Methods

### Patients

From November 2006 to June 2019, patients who 1) had a preoperative diagnosis of lung cancer, 2) met the indications for surgery, 3) and with histologically confirmed SCLC after surgical resection were collected from the Department of Thoracic Surgery, Fudan University Shanghai Cancer Center (FUSCC), Shanghai, China. A total of 157 patients were enrolled. Following clinicopathologic characteristics were prospectively collected including gender, age, smoking history, ECOG scores, time of surgery, surgery procedures, pathology reports, and postoperative adjuvant therapy regimens. To minimize selection bias, four patients (2.5%) were excluded because they died within 30 days after surgery, as these patients would not have received adjuvant therapy (17). Ultimately, 153 patients were included in the analysis. This study was approved by the Ethics Committee and Institutional Review Boards, and all patients were exempt from an informed consent due to the retrospective nature of the study. Surgical resections were classified as sublobectomy, lobectomy and greater than lobectomy according to the extent of resection. All pathologic sections were re-reviewed by 2 pathologists. The clinical and pathological staging were reevaluated according to the American Joint Committee on Cancer (AJCC) eighth edition TNM and the Veterans Administration Lung Study Group (VALSG) staging systems (23, 24).

### Adjuvant Treatment Modalities

Etoposide with cisplatin for 4 to 6 cycles was the predominant adjuvant chemotherapy regimen. For patients with chest radiation, most of them received 50 Gy in 30 days. Patients with prophylactic cranial irradiation (PCI) most frequently received radiation dose of 25 Gy in 10 days. Since all patients in our cohort who underwent chest radiation or PCI had previously received adjuvant chemotherapy and no patients received radiotherapy alone, we divided the patients into non-adjuvant therapy group, adjuvant chemotherapy group and adjuvant chemoradiotherapy group according to the adjuvant treatment modalities. The adjuvant chemoradiotherapy was defined as receiving chemotherapy followed by radiotherapy after surgery, and radiotherapy including chest radiation, PCI or both.

### Statistical Analysis

Overall survival (OS) was defined as the time from surgery to death from any cause or last follow-up. The overall survival was analyzed using the Kaplan–Meier method, with the log-rank test performed to evaluate survival variances. Cox proportional hazard model was used to identify the independent prognostic factors of surgically resected SCLC patients. To evaluate the survival benefits of different adjuvant treatment modalities, further survival analysis and multivariate analysis stratified by clinicopathologic features were conducted. For the multivariate model, all factors with a p<0.05 in the univariate analysis was included. All tests were bilateral, and statistical significance was set at p<0.05. All statistical analysis was performed using IBM SPSS 26.0 (IBM-SPS Inc, Armonk, NY) and RStudio software version 1.3.1093 (RStudio Inc, Boston, Mass).

## Results

### Patient Characteristic

In total, 153 surgically resected SCLC patients were reviewed (**Table 1**). The majority of these patients are older than 60 (65%), male sex (84%), and ever smokers (76%). Most of them underwent lobectomy by muscle-sparing method. Of all patients, 77 (50%), 59 (39%), 12 (8%), and 5 (3%) patients with stage pT1, pT2, pT3 and pT4, respectively. Postoperative pathologic evaluation revealed 70 (46%) patients without lymph node metastases, while 83 (54%) patients with lymph node metastases. There were 108 (71%) patients with pure SCLC and 45 (29%) patients with combined SCLC. In terms of postoperative treatment modalities, 59 (39%) patients only received adjuvant chemotherapy, 60 (39%) patients received adjuvant radiotherapy after chemotherapy (28 cases of chest radiation, 14 cases of PCI and 18 cases of both chest radiation and PCI), whereas 34 (22%) patients did not undergo any adjuvant therapy after surgery because of preference or the intolerance of side effects. The median follow-up time was 56.6 months (95%CI: 44.43-68.77). Median OS was 60.8 months (95%CI: 32.26-89.33).

**Table 1:** Characteristics and adjuvant therapy regimens.

Clinical Characteristic	LS-SCLS (n = 153) (%)
Gender	
Male	129 (84)
Female	24 (16)
Age	
≥60	100 (65)
<60	53 (35)
Smoking history	
Never Smoker	36 (24)
Ever Smoker	117 (76)
Extent of resection	
Lobectomy	110 (72)
>Lobectomy	32 (21)
<Lobectomy	11 (7)
Pathological T stage	
pT1	77 (50)
pT2-4	76 (50)
Pathological N stage	
pN0	70 (46)
pN+	83 (54)
VPI	
Absent	115 (75)
Present	26 (17)
Unknown	12 (8)
LVI	
Absent	71 (46)
Present	64 (42)
Unknown	18 (12)
Residual tumor	
R0	144 (94)
R1	9 (6)
Histology	
Pure	108 (71)
Combined	45 (29)
Postoperative therapy	
Non-adjuvant therapy	34 (22)
Adjuvant chemotherapy	59 (39)
Adjuvant chemoradiotherapy	60 (39)

Values are presented as n (%).

Unknown: Data was not available.

LS-SCLS, Limited-stage small cell lung cancer; VATS, Video-assisted thoracic surgery; VPI, visceral pleural invasion; LVI, lymphovascular invasion.

### Prognostic Factors for Surgically Resected SCLC Patients

**Figure 1:**
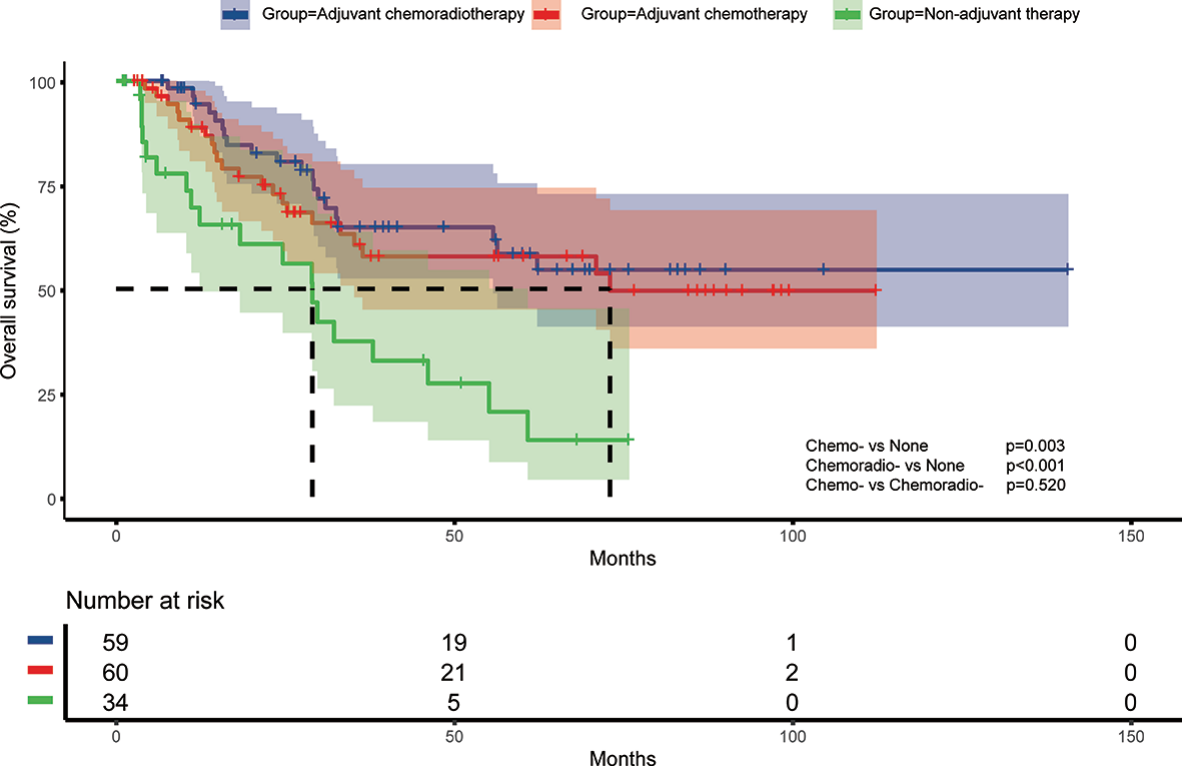
Overall survival of different adjuvant treatment modalities in surgically resected small cell lung cancer patients.

Survival analysis showed both adjuvant chemotherapy group (p=0.003) and adjuvant chemoradiotherapy (p<0.001) had superior prognosis compared with non-adjuvant therapy group (**Figure 1**). Five-year survival rate of non-adjuvant therapy group, adjuvant chemotherapy group and adjuvant chemoradiotherapy group were 20.4% (95% CI: 2.2%-38.6%), 57.9% (95% CI: 43.4%-72.4%), and 58.6% (95% CI: 43.9%-73.3%), respectively. After cox multivariate regression analysis, we found both adjuvant chemotherapy (HR=0.303, 95%CI:0.141-0.654, p=0.002) and adjuvant chemoradiotherapy (HR=0.267, 95%CI:0.122-0.583, p=0.001) were significantly associated with better survival compared with non-adjuvant therapy. In addition, multivariate analysis showed male sex, lymph node metastases, residual tumor, and VPI were associated with poor prognosis (**Table 2**).

**Table 2:** *Cox* regression analysis of factors associated with overall survival for surgically resected SCLC patients (n=153).

Variable	Univariate		Multivariate	
	*P*	HR (95% CI)	*P*	HR (95% CI)
Gender (female *vs* male)	**0.010**	0.264 (0.095-0.730)	**0.003**	0.108 (0.024-0.478)
Age (≥60 *vs* <60)	0.506	1.199 (0.702-2.049)		
Smoking history (never *vs* ever)	**0.027**	0.446 (0.218-0.911)	0.735	0.842 (0.310-2.287)
Operation mode (open *vs* VATS)	0.840	0.930 (0.458-1.887)		
Extent of resection				
Lobe *vs* <Lobe	0.320	2.054 (0.497-8.489)		
>Lobe *vs* <Lobe	0.105	3.356 (0.775-14.536)		
Pathological T stage (pT2-4 *vs* pT1)	**0.031**	1.740 (1.050-2.883)	0.840	1.081 (0.507-2.304)
Pathological N stage (pN+ *vs* pN0)	**<0.001**	2.767 (1.591-4.812)	**<0.001**	3.256 (1.709-6.204)
LVI (present *vs* absent)	**0.002**	2.583 (1.426-4.680)	0.128	1.765 (0.850-3.668)
VPI (present *vs* absent)	**<0.001**	2.986 (1.613-5.526)	**<0.001**	3.730 (1.803-7.718)
Residual tumor (R1 *vs* R0)	**0.022**	2.696 (1.156-6.290)	**0.015**	5.077 (1.369-18.836)
Histologic subtype (combined *vs* pure)	0.628	0.868 (0.490-1.537)		
Adjuvant therapy				
Chemotherapy *vs* None	**0.004**	0.400 (0.215-0.743)	**0.002**	0.303 (0.141-0.654)
Chemoradiotherapy *vs* None	**0.001**	0.328 (0.175-0.616)	**0.001**	0.267 (0.122-0.583)

VATS, Video-assisted thoracic surgery; VPI, visceral pleural invasion; LVI, lymphovascular invasion.

P < 0.05 is indicated by bold.

### Impact of Adjuvant Therapy on Overall Survival

**Figure 2:**
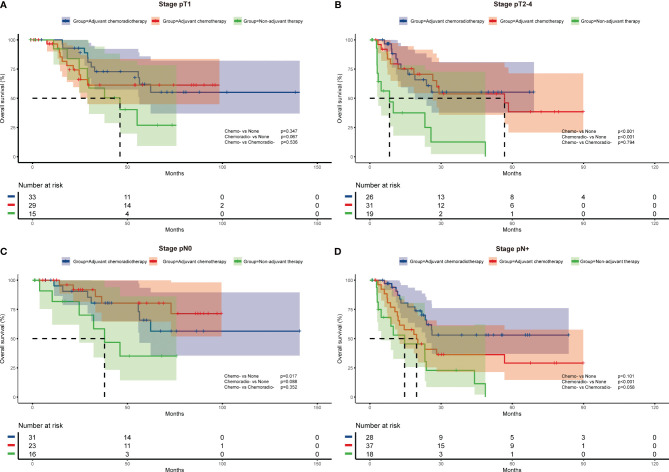
Overall survival of different adjuvant treatment modalities in patients with specific pathologic T stages **(A, B)** and N stages **(C, D)**.

**Figure 3:**
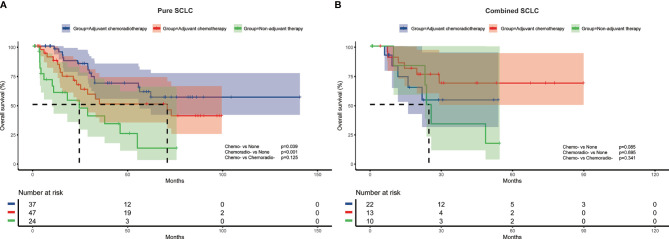
Overall survival of different adjuvant treatment modalities in patients with pure SCLC **(A)** and combined SCLC **(B)**.

To further investigate the impact of adjuvant therapy on survival, we conducted subgroup analyses to assess overall survival stratified by pathologic T stage, lymph node metastasis, and histologic subtypes. For stage pT1 or combined SCLC patients (**Figure 2A** and **Figure 3B**), survival analysis showed overall survival was not significantly different among non-adjuvant therapy, adjuvant chemotherapy, and adjuvant chemoradiotherapy group. However, both adjuvant chemotherapy and adjuvant chemoradiotherapy significantly improved survival for patients with stage pT2-4 (p<0.001 and p<0.001, **Figure 2B**) and pure SCLC (p=0.039 and p<0.001, **Figure 3A**). Meanwhile, survival analysis revealed adjuvant chemotherapy group and adjuvant chemoradiotherapy group had superior survival for pN0 and pN+ subgroups, respectively (p=0.017, **Figure 2C** and p<0.001, **Figure 2D**).

Cox multivariate regression analysis revealed consistent results with survival analysis. After multivariate analysis, both adjuvant chemotherapy and adjuvant chemoradiotherapy were associated with better survival for pT2-4 (HR=0.176, 95%CI:0.053-0.578, p=0.004; and HR=0.115, 95%CI: 0.033-0.405, p=0.001) and pure SCLC patients (HR=0.182, 95%CI: 0.067-0.494, p=0.001; and HR=0.181, 95%CI: 0.071-0.465, p<0.001). For patients with pN0 disease, adjuvant chemotherapy was independently associated with survival (HR=0.219, 95%CI: 0.054-0.891, p=0.034), while adjuvant chemoradiotherapy was associated with improved survival for patients with pN+ disease (HR=0.324, 95%CI:0.138-0.760, p=0.010) (**Table 3**)

**Table 3:** Multivariate analysis of factors associated with overall survival for surgically resected SCLC patients with pT2-4 (n=76), histologic pure SCLC (n=108), pN0 disease (n=70) and pN+ disease (n=83).

Variable	pT2-4		pure SCLC		pN0		pN+	
	*P*	HR (95% CI)	*P*	HR (95% CI)	*P*	HR (95% CI)	*P*	HR (95% CI)
Gender (female *vs* male)			0.137	0.323 (0.073-1.430)			0.065	0.314 (0.092-1.074)
Smoking history (never *vs* ever)			0.704	0.823 (0.301-2.250)				
Pathological T stage (pT2-4 *vs* pT1)			0.147	1.715 (0.827-3.554)				
Pathological N stage (pN+ *vs* pN0)	**0.019**	3.218 (1.207-8.581)	0.246	1.645 (0.709-3.814)				
LVI (present *vs* absent)	0.120	2.346 (0.800-6.878)	**0.002**	3.594 (1.575-8.205)				
VPI (present *vs* absent)	0.084	2.210 (0.900-5.425)			**0.048**	4.149 (1.012-17.013)	**0.001**	3.756 (1.711-8.248)
Residual tumor (R1 *vs* R0)	0.877	0.820 (0.066-10.137)					**0.005**	6.607 (1.749-24.951)
Histologic subtype (combined *vs* pure)					0.085	0.253 (0.053-1.212)		
Adjuvant therapy								
Chemotherapy *vs* None	**0.004**	0.176 (0.053-0.578)	**0.001**	0.182 (0.067-0.494)	**0.034**	0.219 (0.054-0.891)	0.261	0.636 (0.289-1.400)
Radio-chemotherapy *vs* None	**0.001**	0.115 (0.033-0.405)	**<0.001**	0.181 (0.071-0.465)	0.149	0.411 (0.123-1.375)	**0.010**	0.324 (0.138-0.760)

VATS, Video-assisted thoracic surgery; VPI, visceral pleural invasion; LVI, lymphovascular invasion. P < 0.05 is indicated by bold.

## Discussion

Up to now, the treatment modality of SCLC is still dominated by platinum-based chemotherapy combined with radiotherapy (8–11). For early-stage SCLC, surgical resection is recommended (8, 11). In our clinical work, we found a proportion of relatively advanced SCLC patients who underwent surgery incidentally had good survival after adjuvant therapy. Thus, based on a retrospective analysis of a relatively large population, we comprehensively investigated the impact of adjuvant therapy on the survival of surgically resected SCLC patients with pI-III stage, and corroborated the survival benefit of adjuvant chemotherapy and adjuvant chemoradiotherapy for these patients. Furthermore, by means of subgroup analyses, we found other interesting results. Both adjuvant chemotherapy and adjuvant chemoradiotherapy were independently associated with superior survival for pT2-4 and pure SCLC patients. For patients without pathologic lymph node metastasis, adjuvant chemotherapy was independently associated with better survival, while adjuvant chemoradiotherapy was significantly associated with improved survival for patients with pathologic lymph node metastasis.

Previous studies have separately evaluated the efficacy of different adjuvant treatment modalities (17–21, 25, 26). Yao et al. found that SCLC patients with stage pN0 and pN1 who received adjuvant chemotherapy after surgery had a longer overall survival compared to surgery alone (HR=0.57, 95%CI: 0.36-0.91, p= 0.019) (19). Yang and colleagues found that among patients with early-stage (pT1-2N0M0) SCLC after surgery resection, those who received adjuvant chemotherapy had better survival compared with non-adjuvant therapy (HR=0.78, 95%CI: 0.63-0.95, p=0.02) (17). Wakeam E et al. demonstrated a survival benefit of adjuvant radiotherapy for both pN1 (HR=0.79, 95% CI: 0.62-1.00, p=0.05) and pN2 (HR=0.60, 95%CI: 0.48-0.75, p<0.001) diseases, whereas there was no survival benefit for pN0 disease (HR=1.05, 95%CI: 0.83-1.34, p=0.68) (21). In the study by Xu and colleagues, PCI had a survival benefit for resected SCLC patients with stage pII (HR=0.54, 95% CI:0.30-0.99, p=0.047) and pIII (HR=0.54, 95% CI:0.34-0.86, p=0.009), but not for stage pI patients (HR=1.61, 95% CI:0.68-3.83, p=0.282) (18). These studies focused on the impact of the specific adjuvant treatment modalities on the survival at specific pathologic stages. In our cohort, 119 out of 153 patients underwent adjuvant chemotherapy. Of these patients, 59 patients only received adjuvant chemotherapy and 60 patients received adjuvant radiotherapy after chemotherapy. Using *Cox* multivariate regression analysis, we found both adjuvant chemotherapy (HR=0.303, 95%CI: 0.141-0.654, p=0.002) and adjuvant chemoradiotherapy (HR=0.267, 95%CI: 0.122-0.583, p=0.001) significantly improved the survival of SCLC patients after surgical resection. This mutually corroborates with the results of several previous studies. In addition, we found male sex, lymph node metastases, residual tumor, and VPI were associated with poor prognosis.

Previous studies suggested that adjuvant chemotherapy has a survival benefit in relatively early-stage patients (17, 19), and chest radiation and PCI tend to have a survival benefit in relatively advanced patients (18, 21, 22). In a previous study, Nicolas Zhou et al. performed a similar grouping, classifying patients into two subgroups, pN0 and pN+, to investigate the effects of adjuvant chemotherapy, PORT, and PCI on RFS in both groups, respectively, and multivariate analysis found that adjuvant chemotherapy significantly prolonged RFS in both groups (HR=0.49, 95%CI: 0.27-0.91, p=0.024 and HR=0.41, 95%CI: 0.18-0.94, p=0.035, respectively) (20). To further investigate the impact of different adjuvant treatment modalities on survival, we conducted subgroup analyses to assess overall survival stratified by pathologic T stage, lymph node metastasis, and histologic subtypes. Survival analysis showed both adjuvant chemotherapy and adjuvant chemoradiotherapy significantly improved survival for patients with stage pT2-4 and pure SCLC. Meanwhile, adjuvant chemotherapy group and adjuvant chemoradiotherapy group had superior survival for pN0 and pN+ subgroups, respectively. Multivariate analysis revealed consistent results with survival analysis. There was no statistically significant survival benefit of adjuvant therapy for patients with tumor diameters less than 3 cm (pT1) compared to greater than 3 cm (pT2-4), which suggested that the role of adjuvant therapy was limited in very early-stage SCLC. Due to its highly aggressive character, SCLC is more sensitive to adjuvant therapy than NSCLC. Therefore, for pure SCLC, there was a survival benefit of adjuvant therapy. In contrast, for combined SCLC with non-small cell component, the effect of adjuvant therapy was less pronounced. In terms of the results of lymph node metastasis status subgroup analysis, we speculate that SCLC is a very aggressive disease with the potential for metastasis in early stage, therefore, even for patients with R0 resection and pN0 disease, systemic chemotherapy is needed. Meanwhile, patients with pN+ disease have a higher degree of disease progression and are more probable to have local spread and distant metastases than patients with pN0 disease. Thus, pN+ patients are more likely to have a survival benefit from additional chest radiation around the resected lesion or PCI on the basis of adjuvant chemotherapy. Hence, the status of lymph node metastases has a significant impact on the selection of adjuvant treatment options for postoperative patients with SCLC.

This study has several limitations. As a single-center retrospective study, selection and institutional bias are inevitable. In our clinical work, to expand the study population, in addition to patients with early limited-stage SCLC (cT1-2N0M0), we also included patients who had a diagnosis of lung cancer before surgery but not suspected of SCLC and underwent surgery incidentally. This is both a strength and a weakness of our study. On the one hand, it allows our study to assist in the selection of adjuvant therapy for patients with postoperative proven advanced SCLC. On the other hand, it also affects the external veracity of this study to some extent and should be noted when generalizing the findings of this study. In addition, 34 patients in our cohort did not undergo any adjuvant therapy after surgery because of preference or the intolerance of side effects, and survival in this group might have been inherently poor, so there may be some bias. To try to address this issue, we further collated the patients’ ECOG scores and compared them between groups receiving different adjuvant treatments and did not find any statistical difference among the groups (**Supplementary Table 1**). This may have avoided this bias to some extent. There were 11 patients with sublobectomy and 9 patients with residual tumor (R1), which may affect the results.

In conclusion, based on the analysis of 153 patients with SCLC after surgical resection, we investigated the effect of different adjuvant treatment modalities. There were survival benefits for adjuvant therapy, with a significant benefit in pT2-4 and pure SCLC patients compared to pT1 and combined SCLC patients, respectively. More importantly, for patients without pathologic lymph node metastasis, there was a survival benefit with adjuvant chemotherapy. However, for patients with pathologic lymph node metastasis, adjuvant chemoradiotherapy was required to achieve a survival benefit. Further prospective studies are needed to validate the results.

## Data Availability Statement

The original contributions presented in the study are included in the article/**Supplementary Material**. Further inquiries can be directed to the corresponding author.

## Ethics Statement

The studies involving human participants were reviewed and approved by Ethics Committee and Institutional Review Board of Fudan University Shanghai Cancer Center. Written informed consent for participation was not required for this study in accordance with the national legislation and the institutional requirements.

## Author Contributions

DL: Conceptualization; Data curation; Formal analysis; Investigation; Methodology; Software; Validation; Visualization; Writing – original draft; Writing – review & editing. CD: Conceptualization; Data curation; Methodology; Software; Validation; Visualization; Writing – original draft; Writing – review & editing. QZ: Data curation; Investigation; Supervision; Validation; Writing – review & editing. FF: Conceptualization; Formal analysis; Methodology; Supervision; Validation; Writing – review & editing. SW: Investigation; Project administration; Supervision; Writing – review & editing. YL: Data curation; Investigation; Project administration; Supervision; Writing – review & editing. HC: Conceptualization; Funding acquisition; Methodology; Project administration; Resources; Supervision; Writing – review & editing. YZ: Conceptualization; Funding acquisition; Investigation; Methodology; Project administration; Resources; Supervision; Visualization; Writing – review & editing. All authors contributed to the article and approved the submitted version.

## Funding

This work was supported by the National Natural Science Foundation of China (81930073, 81972171, 81772466), Shanghai Municipal Science and Technology Major Project (2017SHZDZX01, VBH1323001/026), Shanghai Municipal Key Clinical Specialty Project (SHSLCZDZK02104), Shanghai Technology Innovation Action Project (20JC1417200) and Pilot Project of Fudan University (IDF159045).

## Conflict of Interest

The authors declare that the research was conducted in the absence of any commercial or financial relationships that could be construed as a potential conflict of interest.

## Publisher’s Note

All claims expressed in this article are solely those of the authors and do not necessarily represent those of their affiliated organizations, or those of the publisher, the editors and the reviewers. Any product that may be evaluated in this article, or claim that may be made by its manufacturer, is not guaranteed or endorsed by the publisher.

## Supplementary Material

Supplementary Table 1 ECOG scores of patients with non-adjuvant therapy, adjuvant chemotherapy and adjuvant chemoradiotherapy.

## Abbreviations

AJCC, the American Joint Committee on Cancer; CI, Confidence interval; FUSCC, Fudan University Shanghai Cancer Center; HR, Hazard ratio; LS-SCLS, Limited-stage small cell lung cancer; LVI, Lymphovascular invasion; OS, Overall survival; PCI, Prophylactic cranial irradiation; RFS, Recurrence-free survival; SCLC, Small cell lung cancer; VALSG, the Veterans Administration Lung Study Group; VATS, Video-assisted thoracic surgery; VPI, Visceral pleural invasion.
