# Supplementary material for: Impact of Adjuvant Therapy on Survival in Surgically Resected Limited-Stage Small Cell Lung Cancer
Source: Front Oncol. 2021 Sep 23;11:704517. doi: 10.3389/fonc.2021.704517 (PMC8495161; doi:10.3389/fonc.2021.704517)
Supplement: Supplementary Table 1 — ECOG scores of patients with non-adjuvant therapy, adjuvant chemotherapy and adjuvant chemoradiotherapy. [file Table_1.docx]

**Supplementary table 1: ECOG scores of patients with non-adjuvant therapy, adjuvant chemotherapy and adjuvant chemoradiotherapy.**

| **Demographics** | **Surgery alone**  **N=34** | **Chemotherapy**  **N=59** | **Chemoradiotherapy**  **N=60** | **p** |
| --- | --- | --- | --- | --- |
| ECOG scores |  |  |  | 0.489 |
| 0 | 30 | 54 | 57 |  |
| 1 | 4 | 5 | 3 |  |
